# Flight range, fuel load and the impact of climate change on the journeys of migrant birds

**DOI:** 10.1098/rspb.2017.2329

**Published:** 2018-02-21

**Authors:** Christine Howard, Philip A. Stephens, Joseph A. Tobias, Catherine Sheard, Stuart H. M. Butchart, Stephen G. Willis

**Affiliations:** 1 Conservation Ecology Group, Department of Biosciences, Durham University, South Road, Durham, DH1 3LE, UK; 2 Department of Life Sciences, Imperial College London, Silwood Park Campus, Buckhurst Road, Ascot, SL5 7PY, UK; 3 School of Biology, University of St Andrews, St Andrews, Fife KY16 9ST, UK; 4 BirdLife International, David Attenborough Building, Pembroke St., Cambridge, CB2 3QZ, UK; 5 Department of Zoology, University of Cambridge, Downing St, Cambridge, CB2 3EJ, UK

**Keywords:** migration, climate change, Afro-Palaearctic migrants, distance, duration, stopovers

## Abstract

Climate change is predicted to increase migration distances for many migratory species, but the physiological and temporal implications of longer migratory journeys have not been explored. Here, we combine information about species' flight range potential and migratory refuelling requirements to simulate the number of stopovers required and the duration of current migratory journeys for 77 bird species breeding in Europe. Using tracking data, we show that our estimates accord with recorded journey times and stopovers for most species. We then combine projections of altered migratory distances under climate change with models of avian flight to predict future migratory journeys. We find that 37% of migratory journeys undertaken by long-distance migrants will necessitate an additional stopover in future. These greater distances and the increased number of stops will substantially increase overall journey durations of many long-distance migratory species, a factor not currently considered in climate impact studies.

## 1. Introduction

Globally, populations of many migratory species are declining at rates far exceeding those of their resident counterparts [1,2]. Identifying the cause of these declines is complicated by the dependence of migrant species on multiple locations, including on their breeding and non-breeding grounds, as well as on the stopover sites used during migration [3,4]. Changes in climate [5,6] and habitat [7] are often cited as the primary drivers of population declines. With long-distance migrants spending a significant proportion of their annual cycle on migration [8], the distance and duration of migratory movements may be significant factors influencing the susceptibility of species to these threats [1,9]. Such long-distance migrants have shown steeper population declines than their resident and short-distance migratory counterparts [3,7]. Ecological conditions *en route,* including food availability and predation pressures at stopover sites, along with weather conditions, affect the survival, migratory schedules and reproductive success of migrants [10–12]. Thus, any increases in the number of sites required by species during their annual cycle may place long-distance migrants at increased risk of exposure to spatially heterogeneous threats [13].

Many studies, across a variety of taxa, have related changes in species’ distributions and population sizes in response to recent climate change [14,15]. Forecasts of the responses of European-breeding birds to future climate change consistently indicate significant poleward shifts in breeding ranges [16]. By contrast, future predictions of shifts in these species' African non-breeding ranges are more diverse [17,18], partly because of the varying latitudes of non-breeding ranges for individual species. Predicted changes in breeding and non-breeding ranges will result in increased migratory distances for some species [18]. Given the time and energetic costs associated with migration, and consequent mortality risk during this period of the annual cycle [10,19], identifying those species that may experience the greatest increases to their migratory journeys in future will help to pinpoint taxa at particular risk of future population decline.

Typically, migratory distance is calculated as the great-circle distance between breeding and non-breeding range centroids [9,18,20]. However, this simple calculation obscures the complex variety of between- and within-species migratory movements [21]. Migratory connectivity—the extent to which breeding and non-breeding populations of a species remain connected—varies significantly among species [22,23]. The degree to which birds use the same migration route can fluctuate not only among individuals within a population but also between years for individuals [24], and between spring and autumn for the same individual [25]. If the potential impacts of climate change on migration are to be identified, it is important that this potential variation within species in migration distances is considered.

Rapid, recent improvements in individual tracking-based technology allow for greater insight into migratory routes and journey times [12,26]. However, as these approaches have been limited to larger species (greater than 250 g for GPS tags) and to those intermediate-sized species that return to a site for recapture (down to 12 g for geolocator tags; which excludes most small passerines), we still lack detailed data on wide-scale species- and population-specific movements for the majority of migratory species [9]. Developments in avian flight theory [27,28], however, can be used to estimate species’ flight ranges and migratory capabilities. These simulation models have been tested and validated using both field studies [29] and wind tunnel experiments [30]. Moreover, an advantage of these models is that they can be integrated with projections of future climate and habitat to predict the impact of environmental change on bird migrations.

Here, we examine the potential effect of climate change on the migration distances of 77 species of common European-breeding birds, based on projections of their current and potential future breeding and non-breeding ranges. We use physiological and morphological traits to estimate the species' flight potential (flight range) before individuals would need to stop to refuel. Based on this, we estimate the number of stopovers that individuals of each species require, on average, to travel between their breeding and non-breeding grounds under current and future projected conditions. Combining total flight and refuelling times allows us to estimate the total duration of migratory journeys. Using data from published geolocator studies, we validate our estimates of the time currently taken for individuals of a subset of species to migrate between their non-breeding and breeding grounds. Finally, we use our analyses to identify which bird species are projected to experience the greatest future changes to their migratory journeys.

## 2. Material and methods

### (a) Species data

Species’ distribution data were compiled for migratory passerine and near-passerine bird species breeding in Europe, focusing on 77 migratory species included in the Pan-European Common Bird Monitoring Scheme [31]. We classified these species into two approximately equal groups according to their migratory strategy: (i) 40 species of short-distance migrants, which migrate principally to Europe and North Africa for the non-breeding season; and (ii) 37 species of long-distance migrants, which spend the non-breeding season entirely in sub-Saharan Africa (see electronic supplementary material, table S1 for classifications). Distribution maps were obtained as separate breeding and non-breeding range polygons for each species [32]. The distribution maps of each species' breeding range were intersected with a 0.5° × 0.5° grid (approx. 50 × 50 km) covering Eurasia west of 52°E and the area of Africa north of 20°N. A species was considered present in a 0.5° grid cell if the cell intersected with the species’ breeding range. The non-breeding ranges of migrants were similarly intersected with a 0.5° grid and converted to presence–absence data across Eurasia (west of 52°E) and Africa.

### (b) Species distributions

We modelled the relationship between species' distributions and the mean of four commonly used bioclimatic variables (mean temperature of the coldest month; growing degree days above 5°; annual precipitation and precipitation seasonality) for the period 1950–2000, hereafter referred to as 2000. To achieve this, we used an ensemble modelling framework, combining four widely applied techniques: generalized linear models (GLMs), generalized additive models (GAMs), generalized boosted regression models (GBMs) and random forests (RFs). Separate species distribution models (SDMs) were built for each species’s breeding and non-breeding ranges following published methods [33]. This approach resulted in 40 models (10 sampling blocks × 4 SDMs) for each species's breeding and non-breeding range. For each species, the 40 models were used to predict the probability that a 0.5° × 0.5° grid cell contains suitable climate during the 2000 period and for 12 climate projections—3 general circulation models (GCMs) × 4 representative concentration pathways (RCPs)—for the future time period, 2061–2080 (hereafter referred to as 2070). This was carried out separately for each species’s breeding and non-breeding range. The median suitability of a cell was taken from across the 40 model predictions for each climate scenario, and a threshold (the calculation of which is detailed in electronic supplementary material, appendix S1) applied to convert to binary predictions of presence or absence. Further details of the calculation of bioclimatic variables, the four SDM modelling approaches, the GCMs and RCPs, and the methods used to account for spatial autocorrelation and to perform model projection, can be found in electronic supplementary material, appendix S1.

We compared the projected shifts in both the breeding and non-breeding ranges between the 2000 period and the 12 median projections for the future period (2070), for both short- and long-distance migrants. Using a circular ANOVA [34], we compared the direction of the shifts between breeding and non-breeding ranges, as well as between short- and long-distance migrants. We used *t*-tests to compare the distance of the shifts of breeding and non-breeding ranges between short- and long-distance migrants.

### (c) Migration journeys

To provide a measure of the distance that a species may have to travel during migration, we first selected a cell that was predicted to be occupied by that species in the non-breeding range for the 2000 period. Cell selection was random but weighted by the median climate suitability across the 40 model projections. We repeated this step for the species's breeding range for the 2000 period, to provide start and endpoints for a migratory journey. We make two assumptions in estimating migratory distances and durations: (i) that individuals travel between the two points as quickly as possible (this is akin to spring migration, when individuals have an incentive to return to the breeding grounds rapidly [35]); and (ii) that individuals travel directly from point to point using the shortest great-circle distance (and are able to stop and refuel over land whenever they deplete their on-board resources). We calculated the great-circle distance between pairs of points, using the ‘geosphere’ package in R [36,37]. This process was repeated 1000 times; in addition to the mean of these migration distances, we report the standard deviation to reflect potential variation in migratory journeys for a single species in any period. This process was repeated for each of the 12 future climate scenarios.

### (d) Flight range estimations

We estimated the potential flight range for each of the 77 species using the program Flight v. 1.24 [29] (http://www.bio.bristol.ac.uk/people/pennycuick.htm)*.* Flight range calculations were based on species-specific measures of wing area, wing span and fat-free body mass [27]. Details regarding the collection of these measures, along with the data, can be found in the electronic supplementary material, appendices S1 and S2. For each species we calculated the maximum potential flight range for both the initial migratory journey and subsequent migrations following a refuelling stopover. These two categories were estimated using different calculations due to the different initial fat-loads of birds (see below). The maximum potential flight range before refuelling was defined as the distance an individual could fly before 95% of its fuel reserves were depleted. We also calculated the time it would take each species to fly these distances, based on their typical flight speed. We assumed that migration occurred in still-air conditions and at an altitude of 500 m (air density of 1.17 kg m^−3^); the latter is typical of passerine migration altitudes [38]. Pre-migratory fat fraction data were not available for most of our study species. Instead, we assumed a pre-migration fat load of 30% for all species, based on typical published pre-departure fat-loads for passerines [27,38,39]. We assumed a refuelling stopover duration of 5 days for all species, based on published data [40–42]. Given an average fuel deposition rate of 4% of non-laden body mass per day [38,43], we assumed that birds depart from stopovers with a fat load of approximately 20%. By comparing the total required migration distance with the estimates of pre-migration and post-stopover maximum potential flight range for a species, we calculated the minimum number of stopovers required by an individual to complete the migration between pairs of start and end locations (as described above). See electronic supplementary material, appendix S1 for details on calculation of migration duration and number of stopovers.

The total time taken to migrate comprised four periods: (i) the time taken for an individual to complete the initial migratory flight, (ii) the total time taken to complete flights between stopovers, (iii) the time taken to travel between the last stopover and the final destination and (iv) the time spent at refuelling stopovers. Each species was classified as either a primarily nocturnal or a diurnal migrant (see electronic supplementary material, appendix S2 for classifications). The total number of hours spent flying (summing (i)–(iii) above) was then divided by either nine for nocturnal migrants (the mean number of hours of darkness during spring migrations) or by 15 for diurnal migrants (the mean number of daylight hours during spring migrations). This provided an estimate of the number of days it would take to complete the flight components of the migratory journey, which we then added to the time spent refuelling at stopovers (5 days × the number of stopovers) to provide an estimate of the total duration of the migratory journey.

For those species for which data are available, we compared our estimates of the total migratory distance, duration and required number of stopovers with migratory track data obtained from published geolocator studies. Given the paucity of such studies available for passerine species, we were able to obtain data with an acceptable sample size (*n* ≥ 6) for only eight out the 77 species. We compared our estimates with spring migrations, as these are generally regarded to be more direct than autumn migrations, and hence are more comparable with our straight line estimates of migration [44]. Using a Mann–Whitney test, we compared the observed distance, duration and number of stopovers of spring migrations with a random sample, equal in number to the sample size of the published study, from the 1000 estimates produced when estimating migration distance. We repeated this 1000 times for each species and report the mean test statistics.

We compared migration distances for 2000 and 2070 time periods for both short- and long-distance migrants, and also examined the relative change in migratory distance for these species. Comparisons were made both within species (to compare the 1000 estimates of current and future migration distance for each species) and across all 77 species (to compare mean estimates of current and future migration distance), using *t*-tests in both cases. A linear regression was used to assess the relationship between mean current and mean future migration distance for short- and long-distance migrants. A deviation of the slope of the regression from unity would indicate that future migration distances were projected to change disproportionately for longer- versus shorter-distance migrants. We then assessed changes in the modelled durations of the migratory journey between current and future using *t*-tests. We also used *t*-tests to compare the mean number of stopovers required currently and in future across all 77 species.

To visualize how migration distance and the required number of stopovers might alter under climate change for two typical long-distance migrant species (*Ficedula albicollis*, collared flycatcher; *Sylvia nisoria,* barred warbler), we plotted the predictions of these two species' current and future breeding and non-breeding ranges, and overlaid the central 90% of the 1000 randomly sampled migration journeys at any given longitude for both current and future scenarios. All analyses were carried out in R v. 3.3.1 [36]. For all statistical tests, α is equal to 0.05. Where mean metrics are given, confidence intervals are standard deviations (s.d.) unless otherwise stated.

## 3. Results

### (a) Species distribution models

Species distribution models (SDMs) for both the breeding and non-breeding ranges of all short- (40 species) and long-distance (37 species) migrants showed good model fit (breeding range mean AUC = 0.97, ± 0.02; non-breeding range mean AUC = 0.94, ± 0.04, electronic supplementary material, table S2).

### (b) Modelling current migration and validation

Based on geolocator tracking studies, our estimates of the total duration of the migratory journey for the 2000 period compared favourably with the recorded journey for most (6/8; 75%) of the species (electronic supplementary material, figure S3). For *Apus apus* (*n* = 6 empirical measurements of migratory duration), we significantly under-predicted the duration of migration, predicting a mean migratory journey of 17.8 days (±0.17 days), in contrast to the observed mean migratory duration of 29 days (± 5.9 days). For *Lanius collurio* (*n* = 6) we also significantly under-predicted the duration of migration, predicting a mean migration duration of 49.1 days (±2.1 days), in contrast to the mean observed migration duration of 62.8 days (±6.1 days).

Migration distance data (from tracked individuals) were available for four of the eight species with validation data available (electronic supplementary material, figure S4). For two species, *Acrocephalus arundinaceus* and *Anthus campestris*, our predicted estimates of migratory distance accorded well with the observed data. Migration distances were under-predicted for the same two species for which we under-predicted migration duration. For *Apus apus*, we predicted a mean migration distance of 6313 km (±243 km), compared with the observed mean migration distance of 9208 km (±871 km). For *Lanius collurio* we predicted a mean migration distance of 8369 km (±328 km) compared with the observed mean migration distance of 11 862 km (±372 km).

Four of the eight tracking studies provided data on the number of stopovers required by individuals during migration, the values for which accorded well with our simulations (electronic supplementary material, figure S5). Test statistics for all of the above comparisons can be found in the electronic supplementary material, table S2.

### (c) Potential impacts of future climate change on migration

**Figure 1.**
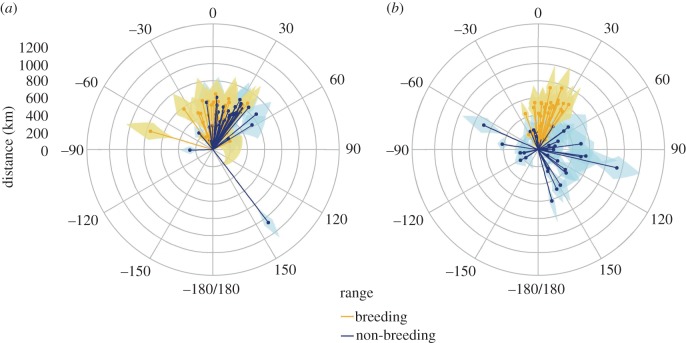
Shifts in the the distance and direction between 2000 and projected 2070 breeding and non-breeding ranges for (*a*) short-distance migrants and (*b*) long-distance migrants. Each line represents a single species. The centre of each polar plot represents the centre of the current (2000) range for each species, taken as the mean of 1000 randomly selected points from the median projection to contemporary climate data. Lines show distance to the centre of the future (2070) range, calculated by taking the mean of 1000 randomly selected points (weighted by probability of occurrence) from the median projection for each of the 12 climate scenarios (3 GCMs × 4 RCPs). Shaded areas represent the standard deviation around the mean range centre from across the 12 different climate scenarios.

We project that, by 2070, the breeding and non-breeding ranges of our 77 study species will have shifted in significantly different directions (circular ANOVA: *F*_153_ = 11.21, *p* < 0.01; figure 1). The projected difference in the direction of range shifts between breeding and non-breeding ranges is particularly pronounced for long-distance migrants (mean breeding range shift = 12.3° [±0.26°], mean non-breeding range shift = 69.8° [±1.48°], circular ANOVA: *F*_73_ = 15.6, *p* < 0.01; figure 1*b*). The difference in the direction of range shifts for short-distance migrants, while also significant, is less pronounced (mean breeding range shift = 7.4° [±0.44°], mean non-breeding range shift = 21.8° (±0.52°), circular ANOVA: *F*_79_ = 5.44, *p* = 0.02; figure 1*a*). The direction of projected breeding range shifts did not differ between short- and long-distance migrants (*F*_76_ = 1.05, *p* = 0.31; figure 1). By contrast, there was a significant difference in the direction of non-breeding range shifts between short- and long-distance migrants (circular ANOVA: *F*_76_ = 10.02, *p* < 0.01; figure 1). The distance by which non-breeding ranges are projected to shift between 2000 and 2070 differ significantly between short- and long-distance migrants (*t*-test: *t*_70.7_ = 3.79, *p* < 0.01). For short-distance migrants we predict a mean non-breeding range shift of 461 km (±164 km), whereas for long-distance migrants we predict a mean non-breeding range shift of 305 km (±195 km). In contrast, we predict that the distance by which breeding ranges will shift between 2000 and 2070 will not differ significantly between short- and long-distance migrants (*t*-test: *t*_73.0_ = 0.12, *p* = 0.90; mean breeding range shift for short-distance migrants = 410 km (±169 km), mean breeding range shift for long-distance migrants = 415 km (±184 km)).

**Figure 2.**
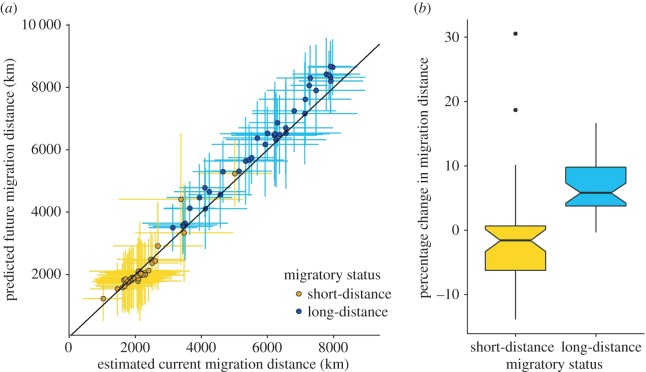
(*a*) Predicted 2000 and 2070 migration distances for 77 European migratory bird species. Mean migration distance is calculated as the mean distance between 1000 randomly sampled points on the breeding and non-breeding grounds. Current distributions are taken as the median probability of occurrence for each grid cell from across 40 predictions (4 SDMs × 10 jackknife iterations) using contemporary (2000) climate data. Future distributions are taken as the median probability of occurrence for each grid cell across all 40 predictions for 12 climate scenarios (3 GCMs × 4RCPs) for 2070. Error bars show the standard deviation around the mean of the 1000 migration distances. The grey line indicates the 1 : 1 line. (*b*) Boxplot showing the percentage change in the mean migration distance between 2000 and 2070 for 40 species of short-distance migrants and 37 species of long-distance migrants.

We project that by 2070, there will be significant increases in the mean migration distances of the 37 species of long-distance migrants, relative to mean estimates for 2000 (paired *t*-test: *t*_36_ = 8.86, *p* < 0.01; figure 2). Specifically, for 86% of long-distance migrants, *t*-tests show significant increases in the estimates of 2070 migration distance compared to the estimates for 2000. For short-distance migrants, we predict both their breeding and non-breeding ranges to shift in broadly similar directions, and thus we predict no consistent overall change in migratory distances (paired *t*-test: *t*_39_ = 0.75, *p* = 0.46; figure 2). For 18% of short-distance migrants, there were significant increases in the estimates of future migration distance relative to current estimates of migration distance. A linear regression of mean 2070 migration distance on mean 2000 migration distance for long-distance migrants produces a slope that differs significantly from 1 (*β* = 1.06, s.e. = 0.04, *t* = 2.29, *p* = 0.03). The same regression for short-distance migrants produces a slope that does not differ significantly from 1 (*β* = 1.08, s.e. = 0.09, *t* = 1.49, *p* = 0.14). This suggests a difference in the proportional change in migration distance in relation to current migration distance and migratory strategy. More specifically, long-distance migrants are projected to increase their relative migration distance more than short-distance migrants (figure 2*b*).

The projected future breeding and non-breeding ranges derived from individual future climate projections did not differ substantially in terms of distribution and extent from the ensemble mean projections. In all future scenarios, all species of long-distance migrants are projected to experience increased migration distances. The 95% quantiles of the mean change in migration distance across all future projections (3 GCMs × 4 RCPs) only overlapped with zero for six out of the 37 species of long-distance migrants (electronic supplementary material, figure S6). This suggests that the projected increases in migration distance for long-distance migrants are consistent, regardless of future climate projections. The variation in potential migratory distances within species (based on the 1000 paired start and end points) between the 2000 and 2070 periods is substantial. For example, only three species of long-distance migrants showed an increase in their migration distance for which the standard deviation around the mean increases did not include zero (figure 2).

**Figure 3.**
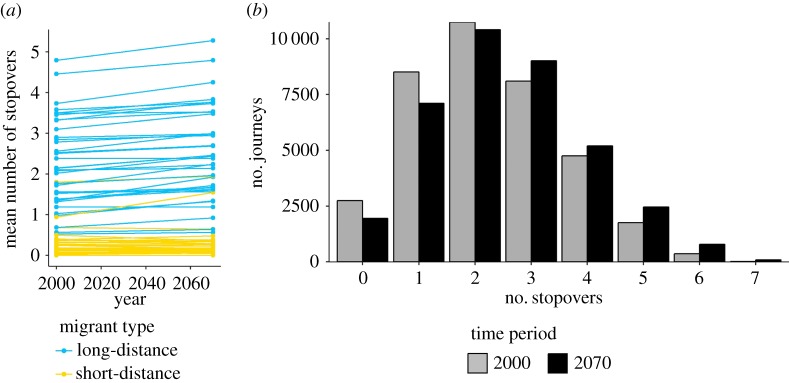
(*a*) Estimated number of required stopovers based on the mean migration distance for 2000 and 2070 projections. Data are shown for 40 species of short-distance migrants (yellow lines) and 37 species of long-distance migrants (blue lines). (*b*) Number of 2000 and 2070 sampled journeys requiring a specific number of stopovers. Data are presented for 37 species of long-distance migrants.

**Figure 4.**
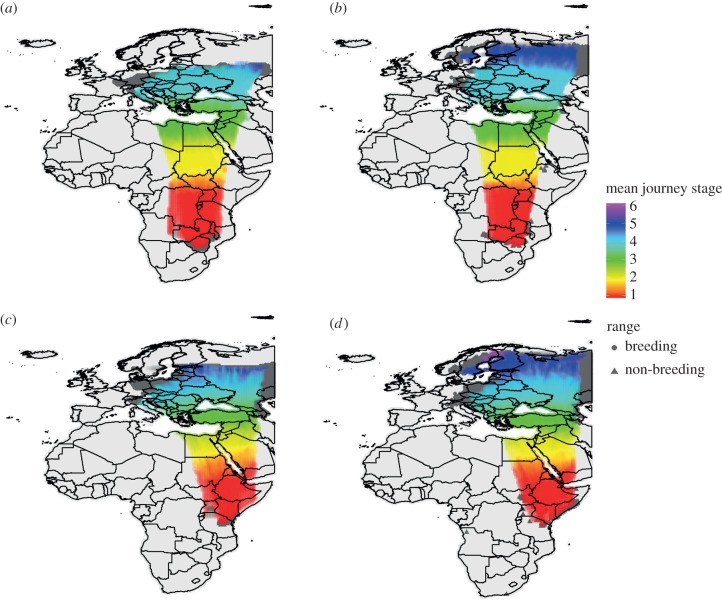
Estimated spring migration routes based on 1000 randomly sampled migratory journeys for (*a*,*b*) *F. albicollis* and (*c*,*d*) *S. nisoria* based on the median (*a*,*c*) 2000 and (*b*,*d*) 2070 occurrence projections. The width of the shaded bars indicates the central 90% of 1000 randomly sampled migration journeys at any given longitude. Colours indicate the mean journey stage of migrant birds passing through a grid cell calculated using both pre-migration and post-stopover maximum flight range distances, with migration starting from the non-breeding range. Dark grey shaded areas indicate breeding and non-breeding ranges.

Overall, based on the modelled migratory distances that need to be covered by long-distance migrants, the mean number of stopovers required is projected to increase significantly by 2070 (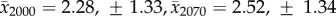, paired *t*-test: t_36_ = 8.98, *p* < 0.01, for ensemble of future climate projections; figure 3). When compared with the mean 2000 estimate of required stopovers for a species, 37% of all future estimated journeys made by long-distance migrants (i.e. from 1000 simulations × 37 species) will require at least one additional stopover compared to current journeys (figure 3*b*). For short-distance migrants, no overall change in required stopovers in future is projected (paired *t*-test: *t*_39_ = 0.04, *p* = 0.97; figure 3). Figure 4 illustrates how we project migratory distances and the required number of stopovers to alter between the 2000 and 2070 time periods.

**Figure 5.**
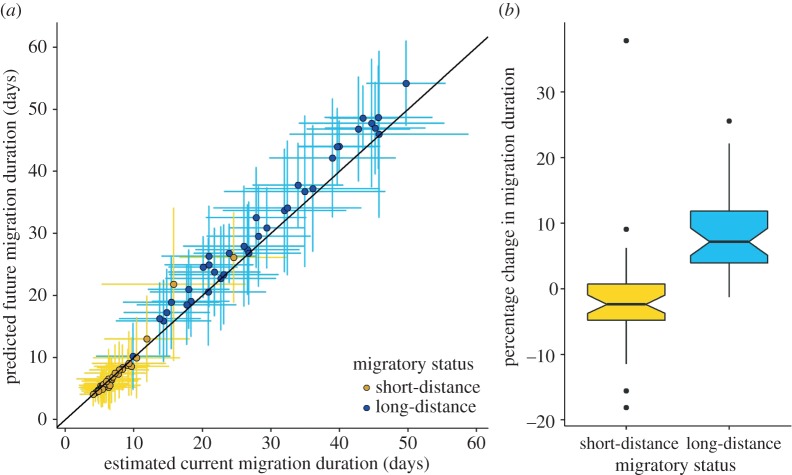
(*a*) Predicted 2000 and 2070 migration duration for 77 European migratory bird species. Mean migration duration is the time taken to migrate the mean migration distance, including time for stopovers. Error bars show the standard deviation around the mean time taken to travel 1000 sampled migration distances. The grey line indicates the 1:1 line. (*b*) Boxplot showing the percentage change in the mean migration duration between 2000 and 2070 for 40 species of short-distance migrants and 37 species of long-distance migrants.

Based on the median range projections to 2000 climate, we estimate that the current journeys of long-distance migrants will take on average 28.8 days (±11.0 days). Based on an ensemble of future projections, we project that the duration of migration for long-distance migrants will increase significantly by 2070 (paired *t*-test: *t*_36_ = 8.91, *p* < 0.01; figure 5), with the mean duration increasing to 31.2 days (±11.6 days). Specifically, there are significant increases between the estimates of future and current migration duration for 84% of long-distance migrant species. By contrast, for short-distance migrants, no overall change in the duration of migration is projected (paired *t*-test: *t*_39_ = 0.11, *p* = 0.91; figure 5), with a current mean duration of 7.2 days (±3.6 days) and a 2070 mean duration of 7.2 days (±4.3 days), based on a median projection and an ensemble projection, respectively. Specifically, *t*-tests comparing the 1000 estimates of current and future migration duration show significant increases in duration for only 5% of short-distance migrant species. Simulation results (mean and standard deviations of migration distance, duration and number of stopovers across the 1000 randomly sampled migration journeys) for each species, model and climate scenario can be found in the electronic supplementary material, appendix S3.

## 4. Discussion

By combining physiological and biological flight models with predicted range changes from species distribution models, we have demonstrated that European long-distance migrant birds are likely to have to spend more time on their migratory journeys in future. Such journeys are predicted to require additional refuelling stopovers, adding to the overall duration of migration. Here, we discuss our findings in relation to the potential impacts of increased migration distances and durations on species, as well as the wider implications of additional stopovers and changes to migratory journeys.

We assessed the consequences of climate change for the migratory process of many species using one of the major global migratory flyways (the Eurasian–African flyway). For over 80% of species that already perform long-distance migration, we project that there will be significant increases in both the distance and time taken to travel between their breeding and non-breeding ranges. One of the greatest predicted increases in migratory distance is for thrush nightingale (*Luscinia luscinia*), which we estimate will have to travel an additional 773 km (±30.3 km) between breeding and non-breeding grounds by 2070. This will add a minimum of five days to the duration of its migratory journey. European bee-eater (*Merops apiaster*) migrations are projected to increase by 1020 km (±19.5 km) and by at least 4.5 days by 2070. A small increase in the duration of the migratory period (i.e. 2–3 days) may not have large impact for some individuals, given the within-species variation in migration duration. Migration, however, is a period of high mortality for birds [10,12,45], potentially as a consequence of increased predation risk [19] or of increased starvation resulting from higher energetic requirements [38,45] and unpredictability in food supply. Any predicted increases to the migratory journey are likely to amplify the exposure of migratory birds to these risks, potentially increasing overall mortality rates and leading to population declines [11].

In addition to the direct costs of migration, altered migration patterns can also have carry-over effects, affecting reproductive success [45]. Recent climate change has led to advancements in the phenology of many species’ life-history events. Longer migrations (in distance and duration) could exacerbate the widely reported effects of phenological mismatch of migrants returning to the breeding grounds [5,6]. Long-distance migrants are particularly susceptible to the effects of this phenological mismatch, with those that have shown the least adjustment in their spring arrival times demonstrating the greatest population declines [6]. For example, despite pied flycatchers (*Ficedula hypoleuca)* advancing egg laying dates by 10 days, this can be insufficient to track spring phenological changes, with a consequent 90% decline in some populations [5]. We predict that, by 2070, this species will take an additional 4 days to travel between the non-breeding and breeding ranges, potentially exacerbating current mismatch. Delays to the arrival of long-distance migrants onto their breeding ranges could further reduce their competitive ability and access to resources, with negative consequences for reproductive success.

Our results indicate that, in future, as many as 37% of journeys made by Afro-Palaearctic migratory species will require an additional refuelling stopover. Conditions at stopover locations can impact not only the migratory performance of birds but also their subsequent reproductive success, by influencing both their timing of arrival and the physical condition in which they arrive at breeding areas [45]. An increased reliance on stopovers might render migratory species more vulnerable to changes in habitat in relatively small and briefly used areas [46]. Furthermore, if the areas where migrants require an additional stopover do not coincide with areas of suitable resource for refuelling, there could be severe consequences for populations.

Detailed knowledge of the habitats and destinations used by birds migrating beyond Europe during the non-breeding season is currently limited, especially in terms of linking specific breeding populations to non-breeding localities. Recent geolocation tracking studies have enhanced our understanding of non-breeding distributions, population-specific migratory pathways and the wide variety of migratory movements demonstrated within some species [23,47–49]. Currently, such data are only available for a few individuals in a small subset of species. Our simulations of migrations, based on the shortest distance between breeding and non-breeding areas, are simplistic, with the routes made by individual migrants in the real world often being more complex. However, by considering intra-specific variation, our models allow for a better understanding of the full range of possible migration routes used by species and the possible impacts of future climate change.

Migration is a naturally plastic trait. Observed changes in migratory behaviour include short-stopping [50] and changes in overwintering locations [51]. For passerines, however, these observations are mostly restricted to short-distance migrants, which already demonstrate a wide range of migratory movements [50]. Long-distance migrants, which tend to have a smaller diversity of migratory movements, may have lower adaptive capacity, rendering them less resilient to environmental change than resident and short-distance migrants [9]. Our models do not currently account for plasticity, but if we are to better understand how migratory species may respond to environmental change, this could be a key area for development.

The evidence from tracking studies of individual birds generally supported the results from our migration models. At present, the statistical power of these analyses is relatively low, given the paucity of available tracking data, but with further technological advancements this sampling should rapidly improve in future. In two of eight species assessed, there were significant differences between our estimates of the total distance and duration of the migratory journey and observations from individual tracking data. Both of these species follow pronounced non-linear migrations, diverting their spring migrations from Africa through Saudi Arabia and West Africa, respectively, before returning to Europe [8,52]. These detours suggest some ecological advantage for deviating from the shortest straight line route, perhaps to avoid crossing extensive ecological barriers, such as the Sahara desert, where refuelling is challenging [19]. Alternatively, detours may be favourable if they enable faster refuelling at stopovers resulting, for example, from pulses in resource availability such as mass insect emergences, or if transport costs are reduced by tail winds [19,52]. If migratory species can benefit from enhanced fuel deposition rates, increasing overall migratory performance [42], longer migratory distances may not necessarily result in longer migration durations. Future research could fruitfully add ecological realism to migration models by linking energetic models, such as those used here, with temporal and spatial resource availability, and weather conditions *en route*.

## 5. Conclusion

These findings shed new light on the likely impacts of climate change on the distance, duration and stopover requirements of long-distance avian migration. They show that, in future, the distances that long-distance migrants will need to travel between suitable breeding and non-breeding habitats will significantly increase. Importantly, by additionally considering the required increase in the number of refuelling stopovers, as well as species-specific flight capabilities, we demonstrate how the overall duration of their journeys is likely to change. We conclude that, in addition to the widely recognized threats of climate and habitat change on species' breeding and non-breeding ranges, migrants will also be exposed to additional pressures from changing migratory journeys, potentially exacerbating anticipated population declines. Our findings add weight to the argument that current climate change impact assessments overlook the complex interplay of spatial and temporal constraints on migratory species, and underestimate their vulnerability to future environmental change. Furthermore, our analyses offer a useful toolkit for more realistic evaluations of the risks faced by the large number of mobile species for which individual tracking data are currently unavailable. Integrating these metrics into future climate change impact assessments could enable more informed conservation actions for migratory species.

## Supplementary Material

Appendix S1

## Supplementary Material

Appendix S2

## Supplementary Material

Appendix S3

## Data accessibility

Additional details of the analysis can be found in the electronic supplementary material, appendix S1. Species morphological data and model simulation results are also available as electronic supplementary material, appendices S2 and S3, respectively.
