# Supplementary material for: Flight range, fuel load and the impact of climate change on the journeys of migrant birds
Source: Proc Biol Sci. 2018 Feb 21;285(1873):20172329. doi: 10.1098/rspb.2017.2329 (PMC5832701; doi:10.1098/rspb.2017.2329)
Supplement: Appendix S1 [file rspb20172329supp1.docx]

# Flight range, fuel load, and the impact of climate change on the journeys of migrant birds

# Supplementary Materials

Christine Howard, Philip A. Stephens, Joseph A. Tobias, Catherine Sheard,

Stuart H.M. Butchart & Stephen G. Willis

Proceedings B, DOI: 10.1098/rspb.2017.2329

Corresponding author: Stephen G. Willis

Email: [s.g.willis@durham.ac.uk](mailto:s.g.willis@durham.ac.uk)

Conservation Ecology Group, Department of Biosciences, Durham University, South Road, Durham, DH1 3LE, UK

Tel: +44 (0)191 3341379

Fax: +44 (0)191 33 41201

List of Supporting Information:

Appendix S1: Methods supplement

Figure S1: Wing morphology of a migratory bird species

Figure S2. Wing area estimations

Figure S3: Comparison of estimated migration durations with geolocator studies

Figure S4: Comparison of estimated migration distances with geolocator studies

Figure S5: Comparison of estimated number of stopovers with geolocator studies

Figure S6: Percentage change in the mean migration distance by 2070.

Table S1: Mean AUC results for the 4 distribution modelling techniques

## Appendix S1: Methods supplement

### Climate data

*Contemporary climate data:* Mean monthly temperature and precipitation data were obtained from Worldclim (Hijmans *et al.* 2005: http://www.worldclim.org) for 1950–2000, a period corresponding to that over which the BirdLife range data were collected. Data were obtained at a 2.5’ resolution. This fine scale grid was overlaid with the same 0.5° x 0.5° grid as used for the species range data. For each half-degree cell, the mean value for each climate variable was calculated from data from all of the Worldclim grid cells within this area. These climate data were then used to calculate four bioclimate values for each half-degree grid cell: mean temperature of the coldest month; growing degree days above 5°; annual precipitation; and precipitation seasonality. Through both direct and indirect effects on vegetation, prey, predators, competition or diseases [2], these variables can limit species ranges and populations. Previously, these variables have been widely and successfully used in models to describe both the breeding and non-breeding range extents [3–7] and abundance patterns of European birds [2,8].

*Future climate projections:* Projections of species distributions were made for the time period 2061–2080 (hereafter referred to by the mid-points, 2070) using data from climate projections from three general circulation models (GCMs) and for four representative concentration pathways (RCPs). These RCPs represent a plausible range of anthropogenic greenhouse gas emission scenarios [9]. Global mean surface temperatures are simulated to increase most by 2100 under the RCP8.5 scenario (3.7°C increase), followed by RCP6.0 (2.2°C increase), RCP4.5 (1.8°C increase) and RCP2.6 (1.0°C). The three GCMs (CCSM4, HADGEM2-ES,MIROC-ESM-CHEM) represent the range in projected climates in the Fifth IPCC Assessment Report [9] and have all been shown to perform well across Europe [10,11]. Projections of mean future climate at a 2.5’ resolution were obtained from Worldclim (Hijmans *et al.* 2005; http://www.worldclim.org) for each of the 12 future climate scenarios (three GCMs x four RCPs) for the time period centred around 2070. These climate projections were used to calculate the four bioclimate variables (described above) for each half-degree cell for the 12 scenarios for the future time period.

### Spatial autocorrelation

Spatial autocorrelation (SAC) occurs when proximate samples show a greater degree of similarity due to distance-related biological processes and spatially structured environmental processes [12]. Failure to account for SAC within SDMs influences both coefficients and inference in statistical analyses through: (1) the violation of the independence assumption and, (2) auto-correlated residuals and hence inflation of type 1 errors [13]. Here, to account for SAC, we utilise a ‘blocking’ method [14], whereby we split the data into ten sampling blocks based on ecoregions (Olson *et al.* 2001; http://www.worldwildlife.org/science/data). Each non-contiguous area of an ecoregion, within the area of study, was classified as a separate sampling unit. These sampling units were then grouped into 10 blocks so that the mean bioclimate was similar across all blocks, but each block covered the full range of bioclimates within the area of study [16].

When fitting a model, nine of the ten blocks were used as the training data set, with model fit assessed using AUC [17,18] on the omitted block. As all blocks cover a similar range of bioclimatic data, this method ensures that a similar range of data was used for both testing and training models, whilst also ensuring that the data are spatially segregated. This method has been shown to perform well at a large scale, minimising the influence of SAC whilst allowing models to capture complex spatial processes [14]. By sequentially omitting each of the ten blocks, fitting the model to the remaining nine blocks and testing the performance on the omitted block, ten models were fitted for each of the four modelling techniques (outlined below). This resulted in 40 models for both the breeding and non-breeding ranges for all 77 species. To assess model fit, the median AUC calculated for the omitted blocks, was taken across the ten models for each of the four modelling techniques for both the breeding and non-breeding ranges for each of the 77 species.

### Species distribution models

### Generalised Linear Models (GLMs)

GLMs [19] were used to fit up to, and including, fourth order polynomial relationships between the three relevant bioclimatic variables and individual species occurrence. For each species, after omitting one sampling block for model evaluation, 81 models (3 bioclimate variables ^ 4 polynomial degrees=81 combinations) were fitted to the remaining nine blocks. AUC was then used to assess the model fit using the excluded block of data. This procedure was repeated excluding each of the ten data sampling blocks sequentially. The combination of polynomial terms for each bioclimatic variable that maximised AUC in each of the ten repeated model fittings was then used to fit a final set of 10 models.

### Generalised Additive Models (GAMs)

Relationships between bioclimate variables and species occurrence were modelled using thin-plate regression splines. Models were fitted to nine blocks of data, after omission of one sampling block for model evaluation using AUC, and the process repeated until each of the ten sampling blocks had been sequentially omitted. These regressions were fitted as a Bernoulli response, using a logit link, and utilised the ‘gam’ function in the ‘mgcv’ R package [20,21].

### Generalised Boosting Methods (GBMs)

Generalised boosting methods, a machine learning technique, builds a large number of simple regression trees, which are then combined to optimise predictive performance [22]. This technique requires the user to set three parameters; learning rate (lr; also known as the shrinkage parameter) determines how much each tree contributes to the final model; tree complexity (tc) controls the number of nodes within a tree; and the number of trees (nt) that are to be retained in the final model. We used a cross validation approach to optimise these parameters for each species. Initially, omitting one block at a time, we fitted a model to the remaining nine blocks using an lr of 0.001, an nt of 5000 whilst allowing tc to vary between 1 and 4. The value of tc that returned the minimum summed error across all blocks from a cross-validation approach was used to fit a final set of 10 models.

### Random Forests (RFs)

Random forests [23,24], are a classification and regression tree (CART) approach, which draws bootstrap samples and a subset of predictors to construct multiple classification trees [25]. This method requires the user to set two parameters; the number of trees (nt) that will constitute the final model and the number of variables randomly sampled as candidates at each split (mtry). We initially set mtry to vary between one and three and then fitted an RF model with 1000 trees to the data after sequentially omitting one block. We assessed the fit of the model on the omitted block using AUC. We then added 500 trees to the model and reassessed AUC. This process was repeated until any improvement in the value of AUC, as a result of the additional trees, was less than 1%. The values of mtry and nt that maximised mean AUC across the 10 blocks of omitted data were used to fit the final 10 models.

### Model Prediction

For each species, the 40 models were used to predict the probability that a 0.5° x 0.5° grid cell contains suitable climate, for the contemporary period and for the 12 climate projections for the future time period (2070). This was done separately for each species’ breeding and non-breeding range. We calculated the median climate suitability of each cell for each species across the breeding range areas (i.e. Europe and North Africa) for the contemporary period, based on the 40 breeding model projections. We used the same approach to assess median climate suitability for each cell across the non-breeding range, but based on the 40 non-breeding model projections across the potential non-breeding area (Europe and Africa, or Africa only for trans-Saharan migrants).

For each species, we applied a threshold to convert median climate suitabilities to binary predictions of presence or absence; the threshold value being set to maximimse the agreement between known and predicted occupied and unoocupied cells (based on maximum kappa) [26]. This was done separately for breeding and non-breeding ranges. We applied these range- and species-specific thresholds to the median climate suitabilities from across the 40 model projections for each of the 12 sets of future projections of modelled climate suitability (3 GCMs x 4 RCPs) for each species, to produce projections of future presence and absence across the breeding and non-breeding ranges for 2070.

### Constraining non-breeding model projections

As similar climate types can occur on either side of the equator, this can result in erroneous projections of non-breeding range in the opposite hemisphere to that which a species currently uses. For this reason, we restricted the areas of Africa over which we projected the non-breeding range, for species that have at least some of their non-breeding range in Africa, as follows. We divided Africa into five latitudinal bands, relating to distance from the equator. One band spanned 11.75 degrees of latitude on either side of the equator (half the distance between the equator and the Tropics of Cancer or Capricorn). One further band on each side of the equator, each of 11.75 degrees, extended from the equatorial band to the Tropics of Cancer or Capricorn. Finally, two bands covered the remaining extent of the continent on either side of the equator. We then projected the non-breeding range of species to those latitudinal bands in which it currently occurs.

**Species biometric data**

Flight range calculations were based on species-specific measures of wing area, wing span and fat free body mass [27]. For 46 species, we obtained measures of wing area from [28]. Published wing area data were not available for the remaining 31 species (see Appendix S2). For these 31 species, we calculated wing area using biometric data compiled from museum specimens. For each species, we measured two key traits from the wings of each species: primary wing chord and secondary length (i.e. primary wing chord minus Kipp’s distance; Figure S1). We sampled 4 adult individuals per species (typically 2 males, 2 females, but including unsexed specimens in some monomorphic species). We note that primary wing chord minus secondary length is equal to Kipp’s distance (see Figure S1), which, in ratio to the wing chord, is a standard measure of dispersal ability in birds [29]. We combined data on primary wing chord and secondary length with published wing span data [29], to derive estimates of wing area using a two-step approach. First, we estimated carpal length (Figure S1) as:

$\text{Carpal length =}\frac{\text{wingspan – (2 * primary wing chord)}}{\text{2}}$ (Eq. 1)

We then estimated wing area using:

$$\text{Wing area=2}\text{ }\text{*}\left[ \left( \text{carpal length}\text{*secondary length} \right)\text{+}\left( \frac{\text{primary wing chord*secondary length}}{\text{2}} \right) \right]$$

(Eq. 2)

To assess the accuracy of our method, we also used these equations to calculate wing area indirectly for 189 European breeding bird species for which wing areas had also been measured directly from physical specimens [28] (including the 46 migrants mentioned above). We found that our approach produced accurate estimates of wing area (least-square regression, r^2^ = 0.96, n = 189; Figure S2), validating its use in calculating wing area for the 31 species lacking direct measurements. Species’ biometric data are provided in Appendix S2.

**Calculating number of stopovers and migration duration**

The number of stopovers was calculated using the following equation:

if (MD < PFR) stopovers = 0

else,

if (MD > PFR) $\text{stopovers=1+floor}\left( \frac{\text{MD-PFR}}{\text{SFR}} \right)$ (Eq. S1)

where MD= migratory distance (Geodesic distance between sampled start and end points), PFR= pre-migratory maximum potential flight range (based on a 30% fat load), and SFR = post-stopover maximum potential flight range (based on a 20% fat load)

Migration duration (assuming 9 hours of flight time per day) was calculated using the following equation:

$\text{Migration duration }\left( \text{days} \right)\text{=}\left( \frac{\text{PFT}}{\text{9}} \right)\text{+}\left[ \left( \frac{\text{S*SFT}}{\text{9}} \right)\text{+}\left( \text{S*5} \right) \right]$ (Eq. S2)

where PFT = flight hours for pre-migratory flight range, S = number of stopovers (from equation S1) and SFT = flight hours for post-stopover flight range.

## **Figures and Tables**


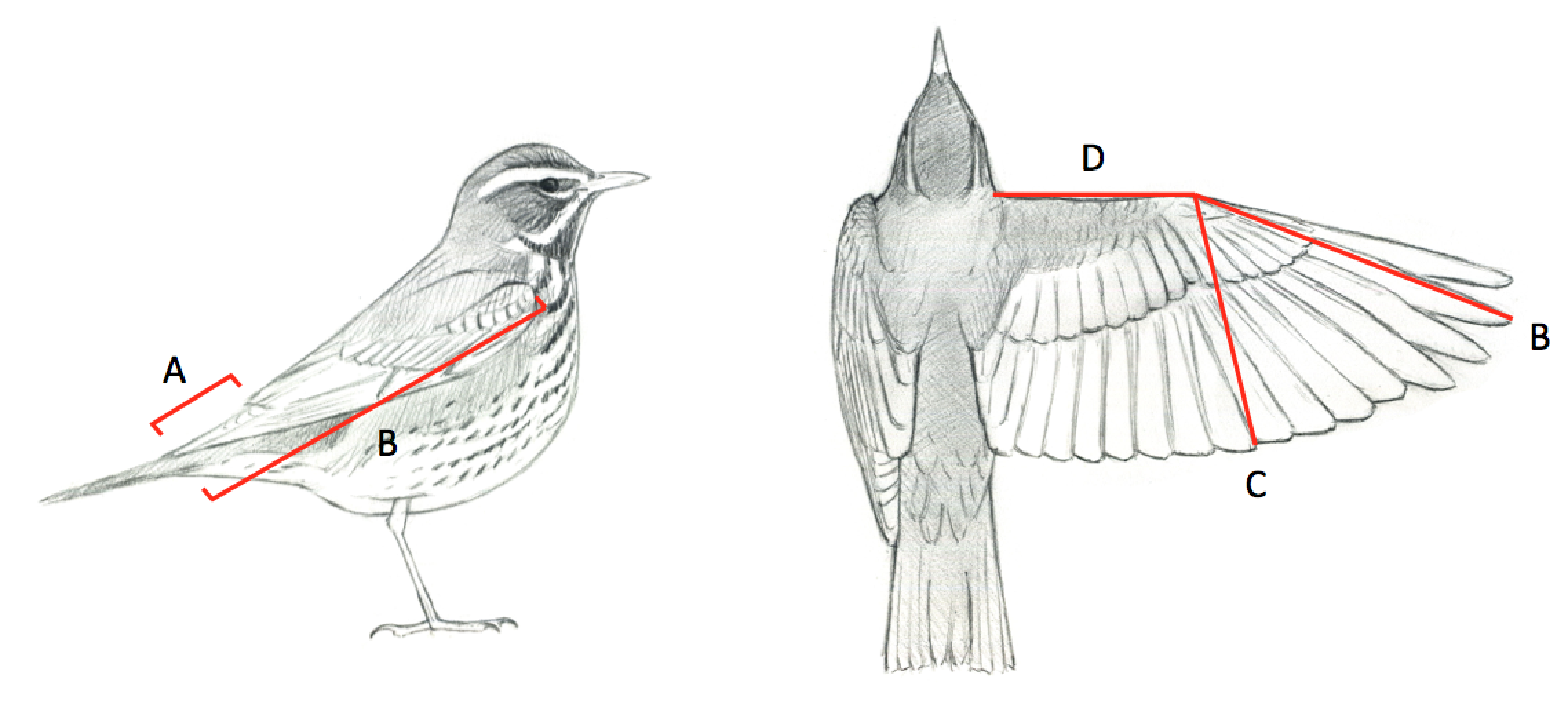


Figure S1: Wing morphology of a migratory bird species (Redwing, Turdus iliacus). Lines indicate biometric variables related to dispersal ability and used in this study to calculate wing area: A, primary extension (Kipp’s distance); B, primary wing chord; C, secondary length; D, carpal length. The wingspan is the total distance between the tips of the two open wings. Illustrations by Richard Johnson.

Figure S2. Wing area estimations from the formulae provided in the main text in relation to measured wing areas provided in [28] for 189 European bird species.


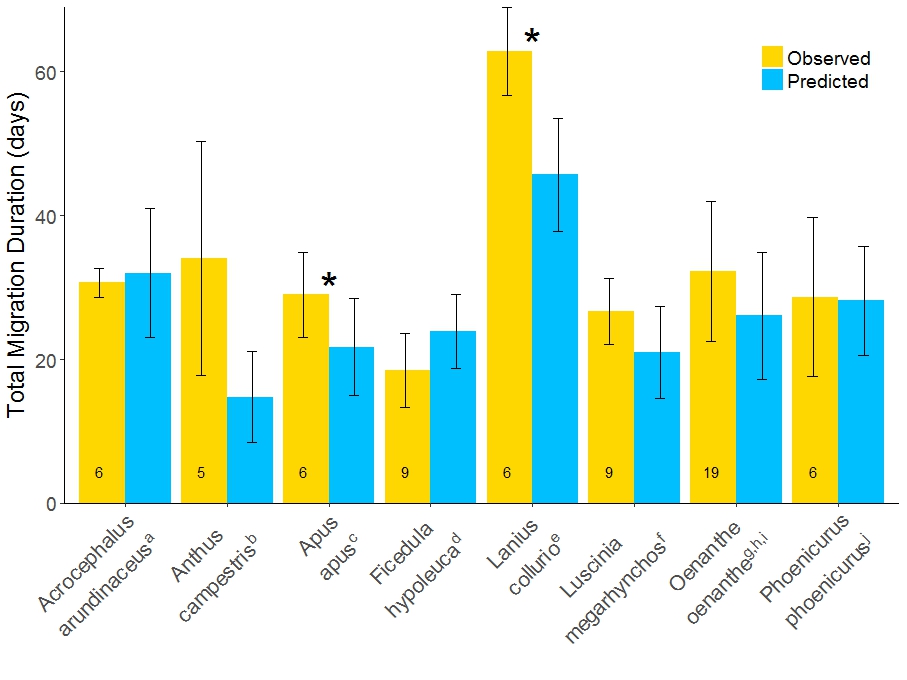


Figure S3: Comparison of mean total migration duration (days) between published geolocator studies of spring migration routes with predictions for eight long-distance sub-Saharan Africa migrants. Estimates are based on the 1000 replicate migrations for each species. Error bars indicate standard deviations around means; numbers on bars indicate sample size; letter codes indicate source publications; a = [30]; b = [31]; c = [32]; d = [33]; e = [34]; f = [35]; g = [36]; h = [37]; I = [38] and j = [39]. Results are combined where more than one study was available *(Oenanthe oenanthe*). Asterisk (*) indicates statistically significant difference between estimated and observed migration duration (p < 0.05). Mean test statistics are reported in Table S2.

**
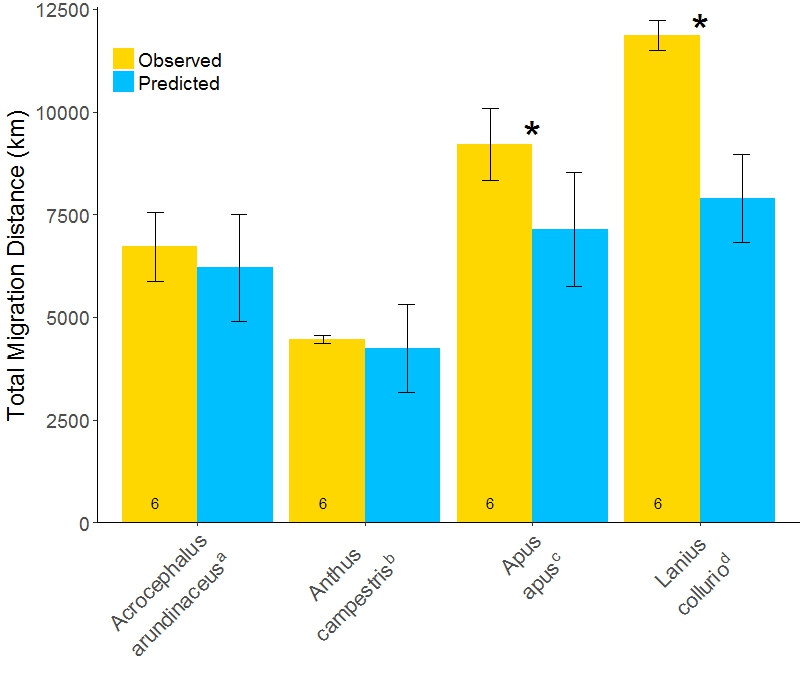
**

Figure S4: Comparison of mean total migration distance (km) between published geolocator studies of spring migration routes with predictions from flight range equations for four long-distance sub-Saharan Africa migrants. Error bars indicate standard deviations around means; numbers on bars indicate sample size; letter codes indicate source publications; a = [30]; b = [31]; c = [32]; and d = [34]. Asterisk (*) indicates statistically significant difference between estimated and observed migration distance (p < 0.05). Mean test statistics are reported in Table S2.

**
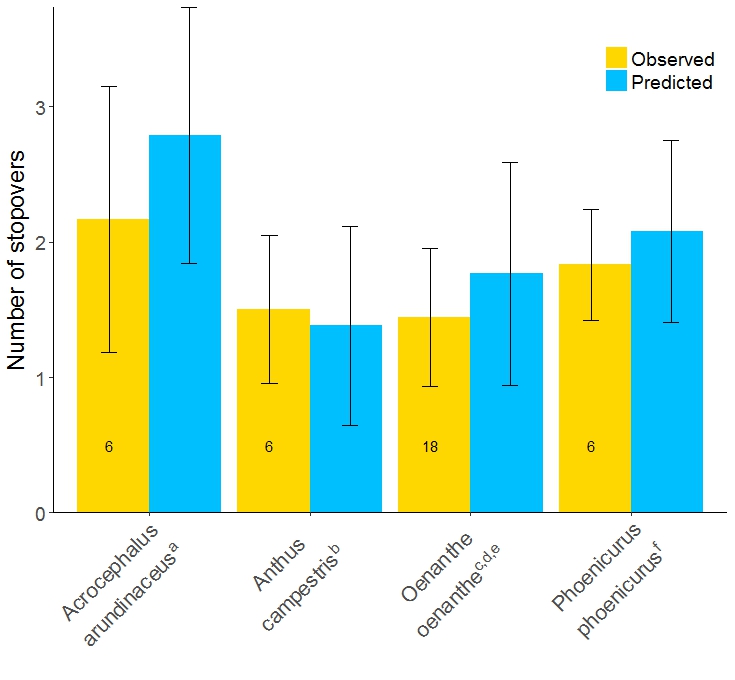
**

Figure S5: Comparison of mean total number of stopovers between published geolocator studies of spring migration routes with predictions from flight range equations for four long-distance sub-Saharan Africa migrants. Error bars indicate standard deviations around means; numbers on bars indicate sample size; letter codes indicate source publications; a = [30]; b = [31]; c = [36], d = [37]; e = [38] and f = [39]. Results are combined where more than one study was available (Oenanthe oenanthe). Asterisk (*) indicates statistically significant difference between estimated and observed number of stopovers (p < 0.05). Mean test statistics are reported in Table S2.


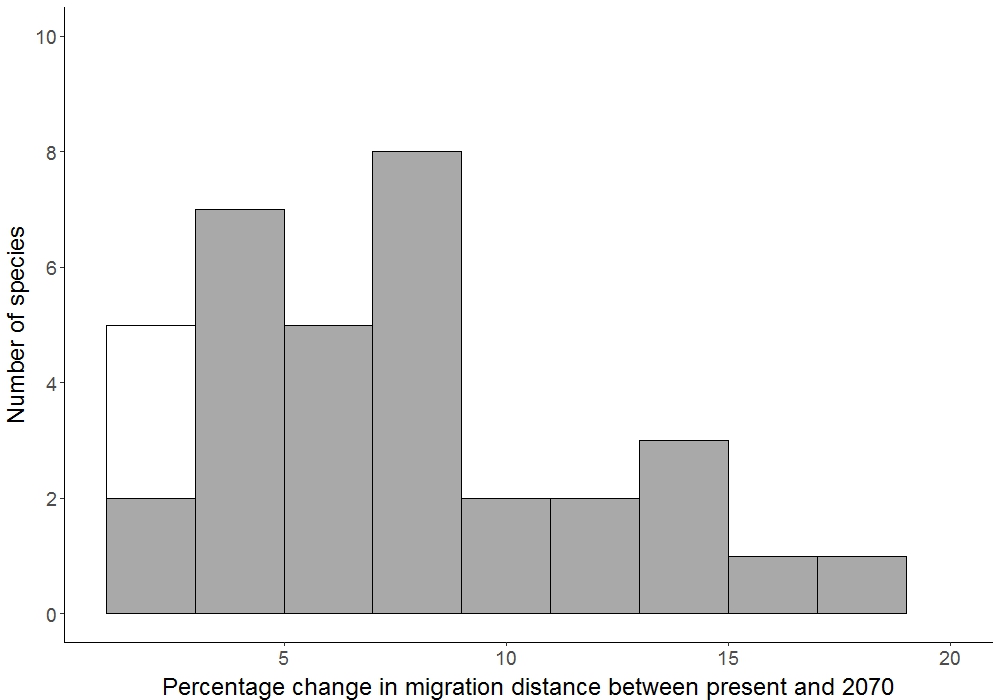


Figure S6: Percentage change in the mean migration distance for 37 long-distance migrants between current and 2070 conditions . The filled portion of each bar represents the proportion of species that are likely to demonstrate the direction of change indicated (i.e. the 95% quantiles of the change in migration distance across the model iterations, GCMs and RCPs did not overlap 0). The open portion of the bars represent those species for which the direction of change indicated was not significant.

Table S1: Mean AUC results for the 4 distribution modelling techniques for the breeding ranges and the non-breeding ranges (77 species in total). AUC values closer to 1 indicate a more accurate model. Figures in brackets indicate standard deviations.

| Modelling Technique | Breeding ranges | Non-breeding ranges |
| --- | --- | --- |
| GLM | 0.963 (± 0.023) | 0.929 (± 0.051) |
| GAM | 0.967 (± 0.019) | 0.934 (± 0.048) |
| GBM | 0.972 (± 0.016) | 0.940 (± 0.044) |
| RF | 0.978 (± 0.013) | 0.958 (± 0.028) |

| Table S2: Mean statistics of 1000 Mann-Whitney tests comparing a random sample from the 1000 migration journey estimates equal in number to the sample size of the published validation studies (N). Results are combined where more than one study was available (Oenanthe oenanthe). | | | | | | |
| --- | --- | --- | --- | --- | --- | --- |
|  | **Species** | **N** | **Mean U-statistic** | **S.D. U- statistic** | **Mean p-value** | **S.D. p-value** |
| Migration Duration | |  |  |  |  |  |
|  | *Acrocephalus arundinaceus* ^[30]^ | *6* | 14.51 | 7.03 | 0.46 | 0.35 |
|  | *Anthus campestris ^[31]^* | *5** | 24.81 | 2.45 | 0.12 | 0.10 |
|  | *Apus apus ^[32]^* | *6* | 32.42 | 2.55 | 0.04 | 0.06 |
|  | *Ficedula hypoleuca ^[33]^* | *9* | 31.07 | 8.50 | 0.08 | 0.12 |
|  | *Lanius collurio ^[34]^* | *6* | 52.71 | 1.41 | <0.01 | <0.01 |
|  | *Luscinia megarhynchos* ^[35]^ | *9* | 61.31 | 8.99 | 0.15 | 0.21 |
|  | *Oenanthe oenanthe* **^[36, 37, 38]^** | *19* | 234.67 | 23.74 | 0.19 | 0.21 |
|  | *Phoenicurus phoenicurus* ^[39]^ | *6* | 18.53 | 4.22 | 0.66 | 0.25 |
|  |  |  |  |  |  |  |
| Migration Distance | |  |  |  |  |  |
|  | *Acrocephalus arundinaceus* ^[30]^ | *6* | 22.43 | 5.66 | 0.48 | 0.31 |
|  | *Anthus campestris* ^[31]^ | *6* | 20.95 | 6.59 | 0.50 | 0.34 |
|  | *Apus apus* *^[32]^* | *6* | 32.52 | 2.86 | 0.04 | 0.07 |
|  | *Lanius collurio* ^[34]^ | *6* | 54.00 | <0.01 | <0.01 | <0.01 |
|  |  |  |  |  |  |  |
| Number of Stopovers | |  |  |  |  |  |
|  | *Acrocephalus arundinaceus* ^[30]^ | *6* | 11.75 | 4.34 | 0.41 | 0.30 |
|  | *Anthus campestris ^[31]^* | *6* | 20.12 | 5.24 | 0.56 | 0.29 |
|  | *Oenanthe oenanthe* **^[36, 37, 38]^** | *18* | 133.53 | 29.06 | 0.33 | 0.29 |
|  | *Phoenicurus phoenicurus*^[39]^ | *6* | 13.48 | 6.32 | 0.43 | 0.30 |
| * Duration data were not available for one individual, however stopover and distance data were | | | | | | |

**References**

1. Hijmans RJ, Cameron SE, Parra JL, Jones PG, Jarvis A. 2005 Very high resolution interpolated climate surfaces for global land areas. *Int. J. Climatol.* **25**, 1965–1978. (doi:Doi 10.1002/Joc.1276)

2. Gregory RD *et al.* 2009 An Indicator of the Impact of Climatic Change on European Bird Populations. *PLoS One* **4**, e4678. (doi:10.1371/Journal.Pone.0004678)

3. Barbet-Massin M, Walther BA, Thuiller W, Rahbek C, Jiguet F. 2009 Potential impacts of climate change on the winter distribution of Afro-Palaearctic migrant passerines. *Biol. Lett.* **5**, 248–251. (doi:DOI 10.1098/rsbl.2008.0715)

4. Doswald N, Willis SG, Collingham YC, Pain DJ, Green RE, Huntley B. 2009 Potential impacts of climatic change on the breeding and non-breeding ranges and migration distance of European Sylvia warblers. *J. Biogeogr.* **36**, 1194–1208. (doi:DOI 10.1111/j.1365-2699.2009.02086.x)

5. Oliver TH, Gillings S, Girardello M, Rapacciuolo G, Brereton TM, Siriwardena GM, Roy DB, Pywell R, Fuller RJ. 2012 Population density but not stability can be predicted from species distribution models. *J. Appl. Ecol.* **49**, 581–590.

6. Bahn V, McGill BJ. 2013 Testing the predictive performance of distribution models. *Oikos* **122**, 321–331. (doi:10.1111/j.1600-0706.2012.00299.x)

7. Barbet-Massin M, Jetz W. 2014 A 40-year, continent-wide, multispecies assessment of relevant climate predictors for species distribution modelling. *Divers. Distrib.* **20**, 1285–1295. (doi:10.1111/ddi.12229)

8. Howard C, Stephens PA, Pearce-Higgins JW, Gregory RD, Willis SG. 2014 Improving species distribution models: The value of data on abundance. *Methods Ecol. Evol.* **5**, 506–513.

9. Stocker TF, Dahe Q, Plattner G-K. 2013 Climate Change 2013: The Physical Science Basis. *Work. Gr. I Contrib. to Fifth Assess. Rep. Intergov. Panel Clim. Chang. Summ. Policymakers (IPCC, 2013)*

10. Brands S, Herrera S, Fernández J, Gutiérrez JM. 2013 How well do CMIP5 Earth System Models simulate present climate conditions in Europe and Africa? *Clim. Dyn.* **41**, 803–817. (doi:10.1007/s00382-013-1742-8)

11. Perez J, Menendez M, Mendez F, Losada I. 2014 Evaluating the performance of CMIP3 and CMIP5 global climate models over the north-east Atlantic region. *Clim. Dyn.* **43**, 2663–2680. (doi:10.1007/s00382-014-2078-8)

12. Dormann CF *et al.* 2007 Methods to account for spatial autocorrelation in the analysis of species distributional data: a review. *Ecography (Cop.).* **30**, 609–628. (doi:DOI 10.1111/j.2007.0906-7590.05171.x)

13. Legendre P. 1993 Spatial Autocorrelation - Trouble or New Paradigm. *Ecology* **74**, 1659–1673. (doi:Doi 10.2307/1939924)

14. Bagchi R *et al.* 2013 Evaluating the effectiveness of conservation site networks under climate change: accounting for uncertainty. *Glob. Chang. Biol.* **19**, 1236–1248. (doi:10.1111/gcb.12123)

15. Olson DM *et al.* 2001 Terrestrial Ecoregions of the World: A New Map of Life on Earth A new global map of terrestrial ecoregions provides an innovative tool for conserving biodiversity. *Bioscience* **51**, 933–938.

16. Moore RT. 2014 blockTools: Blocking, Assignment, and Diagnosing Interference in Randomized Experiments. , R package version 0.6-1.

17. Manel S, Williams HC, Ormerod SJ. 2001 Evaluating presence–absence models in ecology: the need to account for prevalence. *J. Appl. Ecol.* **38**, 921–931. (doi:10.1046/j.1365-2664.2001.00647.x)

18. Brotons L, Thuiller W, Araujo MB, Hirzel AH. 2004 Presence-absence versus presence-only modelling methods for predicting bird habitat suitability. *Ecography (Cop.).* **27**, 437–448. (doi:DOI 10.1111/j.0906-7590.2004.03764.x)

19. Mccullagh P. 1984 Generalized Linear-Models. *Eur. J. Oper. Res.* **16**, 285–292. (doi:Doi 10.1016/0377-2217(84)90282-0)

20. Wood SN. 2011 Fast stable restricted maximum likelihood and marginal likelihood estimation of semiparametric generalized linear models. *J. R. Stat. Soc.* **73**, 3–36.

21. R Development Core Team. 2016 R: A language and environment for statistical computing.

22. Elith J, Leathwick JR, Hastie T. 2008 A working guide to boosted regression trees. *J. Anim. Ecol.* **77**, 802–813. (doi:DOI 10.1111/j.1365-2656.2008.01390.x)

23. Breiman L. 2001 Statistical modeling: The two cultures (with comments and a rejoinder by the author). *Stat. Sci.* **16**, 199–231.

24. Cutler DR, Edwards TC, Beard KH, Cutler A, Hess KT, Gibson J, Lawler JJ. 2007 Random forests for classification in ecology. *Ecology* **88**, 2783–2792. (doi:10.1890/07-0539.1)

25. Prasad AM, Iverson LR, Liaw A. 2006 Newer classification and regression tree techniques: Bagging and random forests for ecological prediction. *Ecosystems* **9**, 181–199. (doi:DOI 10.1007/s10021-005-0054-1)

26. Freeman EA, Moisen GG. 2008 A comparison of the performance of threshold criteria for binary classification in terms of predicted prevalence and kappa. *Ecol. Modell.* **217**, 48–58. (doi:http://dx.doi.org/10.1016/j.ecolmodel.2008.05.015)

27. Pennycuick CJ. 2008 *Modelling the Flying Bird*. Academic Press/Elsevier. See https://books.google.co.uk/books?id=hHsYlQEACAAJ.

28. Bruderer B, Boldt A. 2001 Flight characteristics of birds. *Ibis (Lond. 1859).* **143**, 178–204. (doi:10.1111/j.1474-919X.2001.tb04475.x)

29. Claramunt S, Derryberry EP, Remsen J V, Brumfield RT. 2011 High dispersal ability inhibits speciation in a continental radiation of passerine birds. *Proc. R. Soc. London B Biol. Sci.* (doi:10.1098/rspb.2011.1922)

30. Lemke HW, Tarka M, Klaassen RHG, Akesson M, Bensch S, Hasselquist D, Hansson B. 2013 Annual Cycle and Migration Strategies of a Trans-Saharan Migratory Songbird: A Geolocator Study in the Great Reed Warbler. *PLoS One* **8**, e79209. (doi:ARTN e7920910.1371/journal.pone.0079209)

31. Briedis M, Beran V, Hahn S, Adamík P. 2016 Annual cycle and migration strategies of a habitat specialist, the Tawny Pipit Anthus campestris, revealed by geolocators. *J. Ornithol.* **157**, 619–626. (doi:10.1007/s10336-015-1313-3)

32. Åkesson S, Klaassen R, Holmgren J, Fox JW, Hedenström A. 2012 Migration Routes and Strategies in a Highly Aerial Migrant, the Common Swift Apus apus, Revealed by Light-Level Geolocators. *PLoS One* **7**, e41195. (doi:10.1371/journal.pone.0041195)

33. Ouwehand J *et al.* 2016 Light-level geolocators reveal migratory connectivity in European populations of pied flycatchers Ficedula hypoleuca. *J. Avian Biol.* **47**, 69–83. (doi:10.1111/jav.00721)

34. Tøttrup AP *et al.* 2012 The annual cycle of a trans-equatorial Eurasian–African passerine migrant: different spatio-temporal strategies for autumn and spring migration. *Proc. R. Soc. London B Biol. Sci.* **279**, 1008–1016. (doi:10.1098/rspb.2011.1323)

35. Emmenegger T, Hahn S, Bauer S. 2014 Individual migration timing of common nightingales is tuned with vegetation and prey phenology at breeding sites. *BMC Ecol.* **14**, 1–8. (doi:10.1186/1472-6785-14-9)

36. Arlt D, Olsson P, Fox JW, Low M, Pärt T. 2015 Prolonged stopover duration characterises migration strategy and constraints of a long-distance migrant songbird. *Anim. Migr.* **2**.

37. Schmaljohann H, Buchmann M, Fox J, Bairlein F. 2012 Tracking migration routes and the annual cycle of a trans-Sahara songbird migrant. *Behav. Ecol. Sociobiol.* **66**, 915–922. (doi:10.1007/s00265-012-1340-5)

38. van oosten H, Versluijs R, Van Wijk R. 2014 Migration routes and wintering areas of two Dutch Northern Wheatears Oenanthe oenanthe in the Sahel. *Limosa* **87**, 168–173.

39. Kristensen MW, Tøttrup AP, Thorup K. 2013 Migration of the Common Redstart (Phoenicurus phoenicurus): A Eurasian Songbird Wintering in Highly Seasonal Conditions in the West African Sahel. *Auk* **130**, 258–264. (doi:10.1525/auk.2013.13001)
